# The tissue distribution of nucleobindin-2/nesfatin-1 in the reproductive organs of bitches with regard to the animal’s age and body weight

**DOI:** 10.2478/jvetres-2025-0015

**Published:** 2025-03-25

**Authors:** Marta Rybska, Marek Skrzypski, Karolina Pusiak, Tatiana Wojciechowicz, Adam Mieldzioc

**Affiliations:** Department of Preclinical Sciences and Infectious Diseases, Poznań University of Life Sciences, 60-637 Poznań, Poland; Department of Animal Physiology, Biochemistry and Biostructure, Poznań University of Life Sciences, 60-637 Poznań, Poland; Department of Mathematical and Statistical Methods, Poznań University of Life Sciences, 60-637 Poznań, Poland

**Keywords:** body condition score, canine, nesfatin-1, ovary, uterus

## Abstract

**Introduction:**

Neuropeptide nesfatin-1, a nucleobindin-2 derivative, plays a role in regulating food intake, energy metabolism and body weight. It also interacts with the hypothalamic-pituitary-gonadal axis and has functions in the reproductive system. However, its impact on the canine reproductive tract has not been well documented. This study aimed to investigate the potential role of nesfatin-1 in canine ovarian activity and uterine function.

**Material and Methods:**

Tissue and peripheral blood samples were collected from 60 bitches of various ages and body condition scores (BCS). Analyses included real-time PCR, immunofluorescence examinations and ELISA tests.

**Results:**

Higher level of nucleobindin-2 mRNA were found in the ovarian tissue of both younger and elderly overweight dogs (BCS > 5/9). The elevated expression of nesfatin-1 was observed in the uterine tissues of overweight dogs (BCS > 5/9) compared to its expression in animals in optimal body condition (BCS = 4/9). This finding was consistent with higher nesfatin-1 levels in the peripheral blood of overweight dogs.

**Conclusion:**

The distribution and expression of nesfatin-1 in canine reproductive organs vary depending on the animal’s age and body weight. The role of nesfatin-1 in the reproductive system is influenced by the animal’s body condition and the extent of surplus adipose tissue, which may have significant implications for reproductive functions.

## Introduction

There is growing evidence indicating a positive association between obesity and the occurrence of fertility problems (21). Obese females often experiences hormonal imbalances that lead to menstrual irregularities, anovulation and infertility (42). This association brought the hypothalamic satiety peptide nesfatin-1 under consideration in the treatment of obesity (21) and the regulation of reproductive functions in females (22).

Nesfatin-1 is an 82-amino-acid peptide produced from the posttranslational cleavage of the N-terminal of the N-terminal fragment of nucleobindin 2 (NUCB2) (20). While there is some evidence that nesfatin-1 may interact with G protein–coupled receptors, the nesfatin receptor or receptors remain unknown (25). Initially, it was found that intracerebroventricular (i.c.v.) injection of NUCB2 suppresses food intake, while its expression in the hypothalamic paraventricular nucleus is downregulated under starved conditions (20). Furthermore, it was found that chronic i.c.v. infusion of nesfatin-1 reduced body weight in rats (20). Nevertheless, further studies indicated that the role of nesfatin-1 in the organism may be pleiotropic. For example, nesfatin-1 was found to modulate behavioural functions such as fear or anxiety (35). In addition, nesfatin-1 was found to inhibit adipogenesis (38, 40) and promote glucose-induced insulin secretion (38) and insulin sensitivity (9). These data collectively showed that nesfatin-1 might modulate whole-body energy homeostasis by affecting food intake as well as glucose control and metabolism. It has been theorised that peptides which control appetite and energy homeostasis are also involved in reproductive system functions (9, 12). Therefore, it is not surprising that the potential role of nesfatin-1 in controlling reproduction has been extensively studied. Nesfatin-1 is widely expressed in both male and female reproductive systems, including the testis, epididymis, ovary and uterus. Notably, nesfatin-1 has been identified as a local regulator in reproductive organs (30). It is a regulator of steroidogenesis in the testis and ovary and a controller of the physiological functions of the epididymis and uterus (7, 15, 34). Moreover, the expression of nesfatin-1 was detected in male and female rodents in the hypothalamus and pituitary (36). It was found that nesfatin-1 is co-expressed with gonadotropin-releasing hormone (GnRH) in hypothalamic cells (14). It was demonstrated that i.c.v. administration of nesfatin-1 downregulates hypothalamic expression of GnRH, kisspeptin, follicle-stimulating hormone beta (FSH β) and luteinising hormone beta (LH β) in the pituitary glands of rats (11, 22), and it was shown again to do so specifically in pubertal (45 d) and adult (65 d) male rats (23). Furthermore, nesfatin-1-treated animals had lower levels of testosterone, LH and FSH (23). Additionally, nesfatin-1 was implicated in controlling ovarian steroidogenesis and uterus biology in mice (14). The importance of nesfatin-1 in controlling reproduction in females is supported by human studies showing that the concentration of nesfatin-1 was affected by disorders related to the reproductive system such as polycystic ovary syndrome (PCOS) (41, 43) preeclampsia (44) and gestational diabetes mellitus (16). The majority of data regarding the role of nesfatin-1 was obtained from experiments utilising tissues from rodents, humans and fish (13, 14, 24,10). By contrast, the role of nesfatin-1 in dogs is poorly characterised. While nesfatin-1 expression found to be changed in the uteri of female dogs affected by cystic endometrial hyperplasia (CEH) or pyometra and confirmed overweight, its role and expression pattern in the reproductive organs of healthy bitches are unknown (28). Additionally, other neuropeptides such as phoenixin may contribute to the regulation of the female reproductive system in dogs (26, 27, 28). Phoenixin and nesfatin-1 are similarly expressed in the hypothalamic-pituitary-gonadal (HPG) axis, suggesting a potential interaction between these two peptides (22).

The objective of this study was to examine the potential role of nesfatin-1 in canine ovarian activity and uterine function. The study focused on nesfatin-1 expression and its localisation in the reproductive organs of bitches, taking into consideration their age (young *vs*. elderly) and body condition score (BCS = 4/9 *vs*. BCS > 5/9). Additionally, the study aimed to measure the peripheral blood levels of nesfatin-1 in dogs and explore any possible correlation with the animals’ age and weight.

## Material and Methods

### Animal classification and study material

All study animals underwent a thorough clinical evaluation before routine ovariohysterectomy (OVH), which was performed with the owners’ consent. Selective tissue and blood samples were collected from 60 bitches with either optimal body weight or confirmed excess weight. All OVH procedures were carried out in veterinary clinics in Poznań, Poland. The study was conducted in accordance with the European Convention for the Protection of Vertebrate Animals used for Experimental and Other Scientific Purposes (Directive 86/609/EEC and its revision in Directive 2010/63/EU). Samples were collected from healthy bitches with normal reproductive organs in the dioestrous and early anoestrous phases. Each dog was selected based on its reproductive history, the selection scheme ensuring they represented typical oestrous cycle variations. Animals with any reproductive and metabolic disorders (such as hyperglycaemia or diabetes) and those previously treated with hormone therapy were excluded from the study. The animals were divided into four groups:

A1: dogs up to three years old with optimal body weight and average BCS = 4/9 in a range of 4–5/9,

A2: dogs up to three years old with confirmed excess weight and BCS > 5/9 in a range of 6–8/9,

B1: dogs over six years old with optimal body weight and average BCS = 4/9 in a range of 4–5/9,

B2: dogs over six years old with confirmed excess weight and BCS > 5/9 in a range of 6–8/9.

The oestrous cycle phase was determined through a routine cytological examination and confirmed by measuring the progesterone (P4) concentration in peripheral blood. Detailed information about the studied individuals is presented in Table 1.

**Table 1.** Details of the bitches used in the study

Group	A1	A2	B1	B2
Body condition score	4–5/9	6–8/9	4–5/9	6–8/9
Number of bitches	n = 16	n = 14	n = 16	n = 14
Age (years)				
Max.	3	2.8	12.5	14.0
Min.	1.2	1.0	6.5	6.3
Mean ± standard deviation	2.06 ± 1.39	2.2 ± 0.92	8.3 ± 2.6	10.73 ± 2.34
Body weight (kg)				
Max.	23	35	25	43.5
Min.	2.5	6	4.3	4.2
mean ± standard deviation	8.07 ± 6.73	16.24 ± 7.07	10.5 ± 6.7	19.7 ± 13.71
Breed				
mixed-breed	5	4	4	4
purebred	11	10	12	10
Progesterone (ng/mL)				
Max.	25.93	36.65	39.03	49.98
Min.	1.277	1.439	1.856	1.495
Mean ± standard deviation	6.50 ± 4.30	9.795 ± 8.54	11.70 ± 9.68	12.44 ± 11.74

Following the OVH procedure, the reproductive organs were immediately transported to the laboratory for macroscopic analysis. Only tissues from healthy individuals without visible pathological changes in the reproductive organs were selected for further examination. A fragment of the uterine horn (3–4 cm) and the ovary (about 200 mg) were collected and stored at -80°C for further analysis. Additionally, each animal’s uterine and ovarian tissue samples were fixed in formalin for histopathological and immunofluorescence analysis.

### Histological examination

Macroscopic classification for each study group was confirmed by microscopic histological examination, consistent with our previous description (26, 27). A 2–3 cm fragment of the middle section of the uterine horn was fixed in 10% buffered formalin. Sections obtained were stained using the haematoxylin-eosin (H&E) technique, following procedures described by Rybska *et al*. (27). Tissue sections were assessed using an Axio Lab.A1 microscope (Carl Zeiss Microscopy, Jena, Germany), and the microscope images were analysed using ZEN 3.8 computer software (Carl Zeiss Microscopy).

### Total RNA isolation

Tissue samples (30 mg) from the ovaries and uterus were homogenised with 1 mL of TRI-Reagent solution (Merck, Darmstadt, Germany) using a TissueLyser LT bead homogeniser (Qiagen, Hilden, Germany). Total RNA was extracted following the manufacturer’s protocol (Merck). The quality and quantity of the RNA were measured spectrophotometrically using a NanoPhotometer NP-80 (Implen, Munich, Germany). Only samples with A260/280 absorbance ratios of ~2.0 and A260/230 ratios of 2.0–2.05 were eligible for further analysis. The obtained RNA was stored at –80°C for subsequent reverse-transcription PCR.

### Complimentary DNA (cDNA) synthesis and quantitative reverse-transcription PCR (qRT-PCR) analysis

To prepare cDNA, 1 μg of RNA sample was reverse-transcribed using the Transcriptor First-Strand cDNA Synthesis Kit (Roche Diagnostics, Indianapolis, IN, USA). The protocol included incubation at 65°C for 10 min, incubation at 55°C for 30 min, and final heat inactivation at 85°C for 5 min. The synthesised cDNA separated prepared from all 60 animals was then stored at –20°C for subsequent qRT-PCR analysis. Each reaction was performed in duplicate on a CFX OPUS 384 system (Bio-Rad, Hercules, CA, USA). The primers used, which were for the *Nucb2*-gene encoding nesfatin-1 (17) and for glyceraldehyde 3-phosphate dehydrogenase (*Gapdh*) (26) and β-actin (*Actb*) (26) as endogenous controls, are detailed in Table 2. Each 20 μL reaction contained 10 μL of EvaGreen qPCR Mix (Solis BioDyne, Tartu, Estonia), 1 μL of each primer (5 pmol), 10 ng of cDNA template and distilled water to reach a total volume of 20 μL. The thermal cycling profile was initial denaturation at 95°C for 10 min, followed by 40 cycles of denaturation at 95°C for 10 s, annealing at 60°C for 30 s and elongation at 72°C for 1 s. Melting curve analysis ensured the specificity of amplified products, and negative controls using RNA-free water were included in each run. The efficiency of each reaction and the constitutive expression of reference genes were validated for accurate data interpretation. Relative gene expression levels were quantified using the 2^-ΔΔthreshold cycle (Ct)^ method (32). The calibrator was the mean ΔCt of the six selected samples from the A1 group. The *Actb* mean Ct ± standard deviation (SD) value (in arbitrary units) among all study groups was 21.78 ± 0.29, and for *Gapdh* it was 22.32 ± 0.35. Gene expression data were normalised to the geometric mean of the two reference genes’ Ct and are represented as the ratio of target genes to *Actb+Gapdh*.

**Table 2.** Primer sequences

Gene name	Primer sequence	GenBank accession No. of source gene	Base pairs	Reference
*Nucb2*	F: 5′-CAAGTGATTGATGTGCTGGAA-3′ R: 5′-GCCACTTGTTGTCTTTTCAGTTC-3′	XM_534078	158	(19)
*Gapdh*	F: 5′-GGGTCATCATCTCTGCTCCT-3′ R: 5′-AGTGGTCATGGATGACTTTGG-3′	AF327898.1	150	(26)
*Actb*	F: 5′-CTGGACTTCGAGCAGGAGAT-3′ R: 5′-GATACCGCATGATTCCATCC-3′	AF021873.2	161	(26)

### Radioimmunoassay (RIA)

Measurements of P4 levels in blood plasma samples (n = 40) were made to determine the phase of the oestrous cycle. The levels were measured using an RIA (DIAsource PROG-RIA-CT Kit; DIAsource ImmunoAssays, Ottignies-Louvain-la-Neuve, Belgium) following the manufacturer’s protocol. The P4 concentrations were determined in duplicate within a single series and are presented in Table 1. The standard curve for the P4 RIA assay ranged from 0.19 to 100 ng/mL, and the intra-assay coefficient of variation was 8%.

### ELISA

Nesfatin-1 concentration in blood plasma samples (n = 40) was measured using a commercial Canine Nesfatin-1 ELISA Kit (E0141Ca; BT LAB, Shanghai, China) according to the manufacturer’s protocol. The intra-assay coefficient of variation was 10%. Absorbance values were measured at 450 nm using a Synergy 2 Multi-Mode microplate reader (BioTek, Winooski, VT, USA).

### Immunofluorescence staining

The tissue sections were deparaffinised in xylene twice for 5 min each time and then hydrated using a series of graded alcohol solutions ranging from 100% to 60%. Afterwards, they were boiled three times for 15 min each time in citrate buffer at pH 6.0. Following this, the tissue sections were incubated with a blocking buffer containing 10% rabbit serum and 1% bovine serum albumin (BSA) solution in phosphate-buffered saline (PBS) for 2 h at room temperature. Subsequently, they were incubated with anti-nesfatin-1 antibody (diluted 1:250 in PBS) overnight at 4°C. After a washing procedure in tris buffered saline with Tween 20 (TBST) buffer, the tissue fragments were incubated with Alexa Fluor 555 secondary antibody (Invitrogen, Carlsbad, CA, USA) diluted 1:500 in TBST buffer for 1 h at room temperature. The slides were dried for 10 min at room temperature and then covered with Fluoroshield with 4′,6-diamidino-2-phenylindole for staining nuclei (Sigma-Aldrich, St. Louis, MO, USA). Finally, the tissue sections were photographed using a Zeiss Axiovert 200M fluorescence microscope (Carl Zeiss, Oberkochen, Germany).

Fluorescence intensity (immunoreactivity) was measured using ImageJ/Fiji software (31). A minimum of four different areas per microscopic slide were analysed. The macro area analysed was approximately 100 mm^2^ (from the whole area of the image of 450 mm^2^). From the uterus, the luminal epithelium, endometrial gland and endometrial stroma were analysed separately. From the ovary section, the antral and primary follicles, cortical stroma and corpus luteum cells were analysed. The nesfatin-1 antibody signal was validated in the rat hypothalamus as a positive control. To verify the specificity of the secondary antibody, a negative control was performed using a solution of 1% BSA in 0.1 M PBS equivalent to the primary antibody.

### Statistical analysis

Statistical analysis was conducted using GraphPad Prism version 10.0 (GraphPad Software, San Diego, CA, USA). Data normality was assessed with the Shapiro–Wilk test. Analysis of variance followed by Tukey’s post-hoc test was employed to analyse the expression levels of nesfatin-1 transcript and peptide plasma concentration among the studied groups. The level of nesfatin-1 immunoreactivity among the groups presented abnormal distribution and was analysed using the Kruskal–Wallis test, followed by Dunn’s post-hoc test. Results are expressed as mean ± SD, with statistical significance set at P-value < 0.05. The correlation coefficient (r) was compared to determine the relationship between nesfatin-1 blood plasma concentrations and the animals’ age and BCS.

## Results

### Expression of *Nucb2* in ovarian and endometrial tissues

**Fig. 1.**
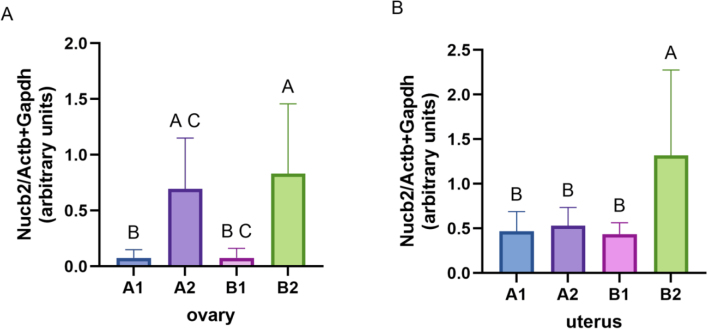
Relative expression of *Nucb2* in A – the canine ovary (A) and B – the uterus normalised to the mean expression of the *Actb* and *Gapdh* housekeeping genes. Bars represent mean ± standard deviation. A, B and C – significant differences between groups (P-value < 0.05). A1 – younger bitches with optimal body weight (body condition score (BCS) = 4/9); A2 – younger confirmed overweight bitches (BCS > 5/9); B1 – elderly bitches with optimal body weight (BCS = 4/9); B2 – elderly confirmed overweight bitches (BCS > 5/9)

*Nucb2* expression was found to be significantly higher in the ovarian (Fig. 1A, P-value < 0.05) and endometrial tissue (Fig. 1B, P-value < 0.05) collected from elderly overweight bitches (BCS > 5/9 – the B2 group) compared to those with normal body weight (BCS = 4/9 – the A1 and B1 groups). There were no significant differences (Fig. 1B, P-value > 0.05) in the expression of *Nucb2* mRNA in the endometrium between younger animals with different BCS (between the A1 group and the A2 group). However, in ovarian tissue, significant differences (Fig. 1A, P-value > 0.05) were shown between those groups (A1group vs. A2 group). These results suggest that as dogs age and accumulate fat, their metabolic rates may slow down, leading to increased expression of nesfatin-1 in reproductive tissues.

### Concentrations of nesfatin-1 in canine blood plasma

**Fig. 2.**
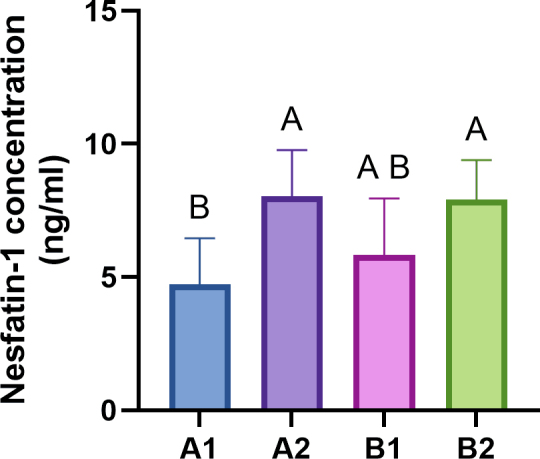
Concentrations of nesfatin-1 in peripheral blood from healthy bitches in optimal body condition (A1 and B1 groups) and overweight bitches (A2 and B2 groups). Bars represent the mean ± standard deviation. A and B – significant differences between groups (P-value < 0.05). A1 – younger bitches with optimal body weight (BCS = 4/9); A2 – younger confirmed overweight bitches (BCS > 5/9); B1 – elderly bitches with optimal body weight (BCS = 4/9); B2 – elderly confirmed overweight bitches (BCS > 5/9)

The plasma NUCB2/nesfatin-1 concentrations in the healthy bitches are shown in Fig. 2. The nesfatin-1 concentrations were significantly increased in the blood of dogs with confirmed excess weight (BCS > 5/9) in both the A2 and B2 groups compared to younger animals with ideal body condition (BCS = 4/9, A1 group) (P-value < 0.05). Pearson’s correlation coefficients were select to examine the relationship between nesfatin-1 concentration, BCS and animal age across all study groups. The results, presented in Table 3, show a positive correlation between nesfatin-1 concentration and BCS (r = 0.5875, P-value < 0.001). However, the correlation between nesfatin-1 concentration and animal age was not statistically significant (P-value > 0.05).

### Immunolocalisation and immunoreactivity of nesfatin-1 in canine ovary and uterus sections

**Fig. 3.**
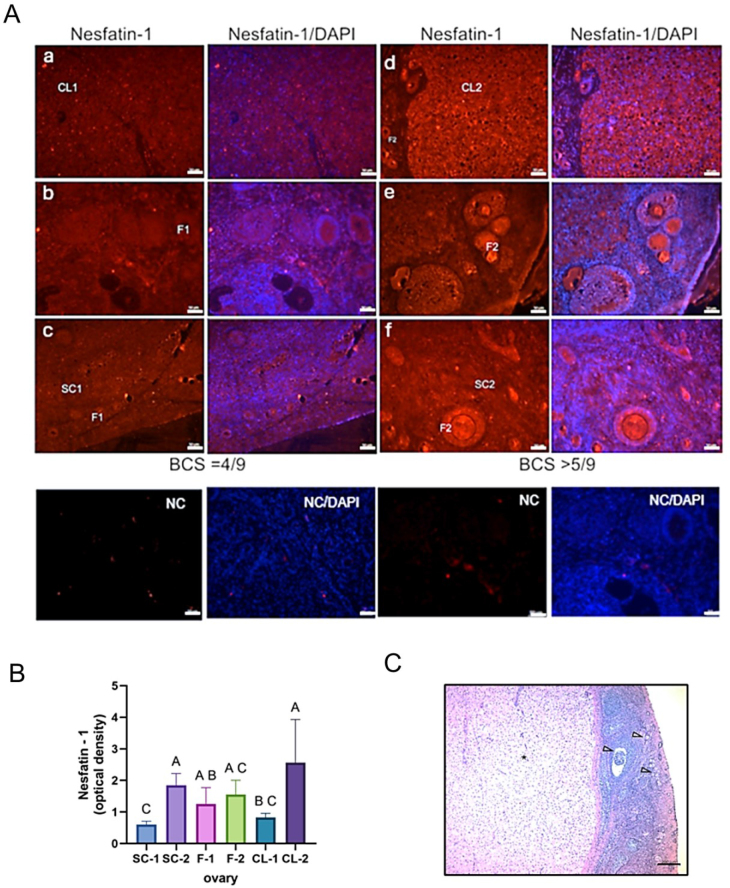
A – Representative canine ovary sections showing nesfatin-1 localisation (in red) and cell nuclei stained by 4′,6-diamidino-2-phenylindole (DAPI – in blue). Signal detection of nesfatin-1 in the ovaries of bitches with body condition score (BCS) = 4/9 (a, b and c) and overweight bitches with BCS > 5/9 (d, e and f). Scale bar – 50 μm. NC – negative control; B – Level of nesfatin-1 immunoreactivity analysed in a group of bitches with BCS = 4/9 and overweight bitches with BCS > 5/9. CL – corpus luteum; F – ovarian follicles; SC – ovarian cortical stromal cells. Bars represent the mean level of neuropeptide immunoreactivity ± standard deviation. A, B and C – statistically significant difference between groups at P-value < 0.05. SC-1, F-1 and CL-1 – dogs with BCS = 4/9; SC-2, F-2 and CL-2 – dogs with BCS > 5/9. C – haematoxylin-eosin staining in a representative photomicrograph of an ovarian section from healthy female dogs in the dioestrous phase; arrows – ovarian follicles; asterisk – corpus luteum. Scale bar – 200 μm

**Fig. 4.**
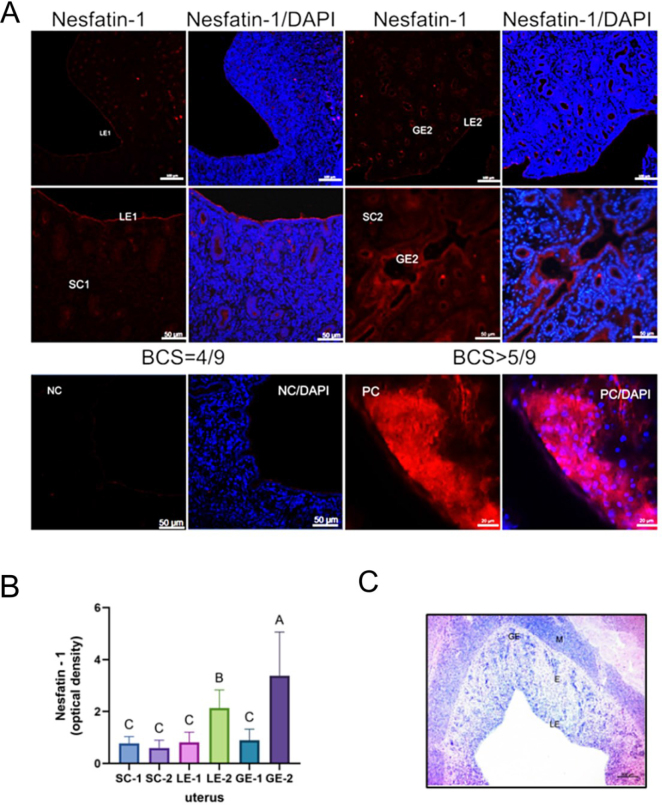
A – Representative canine uterus sections showing nesfatin-1 localisation (in red) and cell nuclei stained by 4′,6-diamidino-2-phenylindole (DAPI – in blue). Signal detection of nesfatin-1 in the uteri of bitches with body condition score (BCS) = 4/9 (a and b) and overweight individuals with BCS > 5/9 (c and d). Scale bar – 100 μm (a and c), 50 μm (b, d and negative control (NC)), 20 μm (positive control (PC)). B – Level of nesfatin-1 immunoreactivity in the endometrial epithelium (LE), the glandular epithelium (GE), and endometrial stromal cells (SC). Bars represent the mean level of neuropeptide immunoreactivity ± standard deviation. A, B and C – statistically significant difference between groups at P-value < 0.05. LE-1, GE-1 and SC-1 – dogs with BCS = 4/9; LE-2, GE-2 and SC-2 – dogs with BCS > 5/9. C – Representative photomicrograph of histological staining with haematoxylin and eosin of a uterine section from a healthy female dog in the dioestrous phase; M – myometrium; E – endometrium; Scale bar – 200 μm

The nesfatin-1 signal was primarily localised in the ovarian cortical stromal cells, corpus luteum and both secondary and primary ovarian follicles (Fig. 3A). In uterine tissue, nesfatin-1 fluorescence was observed in the glandular epithelium, endometrial stroma and luminal epithelium (Fig. 4A).

Immunoreactivity analysis showed a higher nesfatin-1 signal in the ovaries of overweight animals (BCS > 5/9). A significantly higher nesfatin-1 signal was noted in the ovarian stroma (P-value < 0.05) and corpus luteum (P-value < 0.05) of these animals than in this tissue of dogs with normal body condition (BCS = 4/9) (Fig. 3B). In uterine tissue, nesfatin-1 immunoreactivity as measured by optical density was also significantly higher in overweight bitches than in those with ideal body condition (Fig. 4B). Significantly greater nesfatin-1 immunoreactivity was observed in the endometrial epithelium (P-value < 0.05) and glandular epithelium (P-value < 0.05) in overweight individuals compared to animals in normal body condition (Fig. 4B). The localisation of nesfatin-1 and its immunoreactivity (optical density) in tissue sections from younger and elderly dogs were similar, without any significant difference (the results are not shown). Representative photomicrographs of H&E-stained sections of the reproductive tracts are shown in Figs 3C and 4C. Histological analysis of uterine and ovarian sections confirmed the image of the reproductive organs to be typical without any inflammatory alterations. The image was characteristic of the dioestrous and anoestrous phases.

## Discussion

Recent studies have shown that nesfatin-1, along with various neurotransmitters regulating the HPG axis, is responsible for neuroendocrine regulation and stimulates responses to pain (29), stress (42), food intake (29, 40) and reproductive function (15, 23, 26). These findings of this research on bitches suggest that nesfatin-1 could impact the homeostasis of the reproductive system and metabolic changes in bitches. This study examined the expression and localisation of nesfatin-1 in the ovarian and uterine tissue of young and older bitches, as well as those with normal body conditions and confirmed excess weight.

The results indicated a potential link between metabolic alterations and reproduction with elevated nesfatin-1 expression. They showed higher expression of nesfatin-1 in the ovarian and uterine tissues of older bitches and those with greater body weight (BCS > 5/9) than in these tissues of healthy animals in optimal body condition. These results suggest that ageing and increased body fat in female dogs are associated with metabolic changes that may reduce their reproductive potential.

The peptide nesfatin-1 is believed to influence the regulation of energy homeostasis in the ovaries and steroidogenesis. Several studies confirmed that *Nucb2* mRNA and nesfatin-1 are expressed more in the ovary (3, 13, 14) than in the hypothalamus (13). Ovarian nesfatin-1 expression varies with the oestrous cycle, peaking during oestrus (23). It is responsive to ovarian activity, showing low levels in hyperactive ovaries and high levels in regressed ones (3). Nesfatin-1 is found in follicle cells, theca and granulosa cells, and interstitial cells (3). A study by Cao *et al*. (4) showed that *Nucb2* transcript levels decrease as large ovarian follicles develop. Additionally, studies in zebrafish showed that nesfatin-1 inhibited oogenesis and suppressed oocyte maturation. Intraperitoneal nesfatin-1 injection reduced ovarian mRNA expression of LH and FSH receptors (4) and increased gonadotropin-inhibiting hormone expression (5). Nesfatin-1 in granulosa cells supported progesterone secretion and cell proliferation and decreased circulating β-oestradiol levels (5). However, as mentioned in the introduction, it is important to note that a proven identification of the nesfatin-1 receptor has not yet been made, complicating research into the mechanisms through which this neuropeptide affects the body.

The presented results confirmed the possible role of nesfatin-1 in canine ovary regulation. We identified elevated nesfatin-1 signals in the ovarian stroma and corpus luteum cells of elderly female dogs with a BCS greater than 5/9. In the ovarian follicles of the overweight dogs in the dioestrus phase, a stronger nesfatin-1 signal was observed in the theca and granulosa cells, while a weaker signal was detected in the oocytes. These findings are in line with those of a prior study on mice ovaries (13, 14, 15). In them, nesfatin-1 was expressed in the theca cells, interstitial cells, granulosa and cumulus cells around the oocytes. However, the nesfatin-1 signal of corpus luteum staining was very weak. The authors suggested that nesfatin-1 may play a local regulator role in steroidogenesis and energy homeostasis in the ovary (15).

In our results, significantly higher levels of nesfatin-1 peptide were observed in the uteri of overweight bitches, particularly in the luminal and glandular epithelium, compared to these levels in dogs with normal body conditions (BCS = 4/9). This finding aligns with those of previous studies on nesfatin-1 in the female reproductive tract. The immunoreactivity of NUCB2/nesfatin-1 was observed in endometrial epithelial cells, endometrial glands (14) and oviduct cells (37). Furthermore, nesfatin-1 was expressed at higher levels in the uterus than in the hypothalamus (14). The binding sites of this neuropeptide have been identified on neutrophils, which play a key role in endometrial regeneration. This suggests that nesfatin-1 may attract neutrophils from the bloodstream to the endometrium and activate them (15). However, a possible role of nesfatin-1 in uterine secretion requires further investigation. Its expression is influenced by the oestrous cycle, peaking during oestrus (22), and may be stimulated by 17β-oestradiol administration (37). Additionally, NUCB2/nesfatin-1 was present in the amnion and decidua of the placenta, and was found in the syncytiotrophoblast throughout all trimesters of pregnancy (8).

Gonadal hormones may regulate serum levels of nesfatin-1. Research on the subject showed that the serum concentration of nesfatin-1 was regulated by oestradiol and progesterone hormones in female albino rats, with the effect of oestradiol being more prominent than that of progesterone. In male albino rats, this peptide regulation was influenced by testosterone. The nesfatin-1 concentration significantly dropped in individuals after orchidectomy or ovariectomy, but its serum levels increased after sex hormone injection in rats of both sexes (12). In our previous study, we examined the possible connection between serum nesfatin-1 and progesterone concentration in bitches. We observed a positive correlation; however, not one for which the data demonstrated statistical significance (28).

The possible relationship between serum nesfatin-1 levels and body mass index (BMI) invites various conclusions. Some research indicated that nesfatin-1 concentrations has a negative association with obesity and BMI (1). Conversely, other studies have found a positive correlation between nesfatin-1 levels and BMI, body weight, and fat mass in people with obesity or metabolic syndrome (24).

Our results in bitches have revealed a positive association between nesfatin-1 expression in reproductive tissues and BCS. Both young and elderly overweight dogs showed elevated nesfatin-1 levels in their blood compared to younger dogs with an ideal BCS of 4/9. These findings suggest that nesfatin-1 may affect metabolic regulation in older bitches, especially as their metabolic rate decreases and body weight increases with age. This result implies a potential role for nesfatin-1 in a dog’s adipogenesis and fat tissue metabolism.

In addition to being correlated with reproductive disorders, NUCB2/nesfatin-1 has been noted to be involved in the development of endometriosis (33), endometrial cancer (18, 39) and ovarian cysts (2, 6, 41). While some studies report high NUCB2/nesfatin-1 expression in endometrial cancer (39), others show low serum nesfatin-1 levels in endometriosis (33). Moreover, nesfatin-1 may interact with phoenixin-14 in regulating the canine reproductive system. Our results indicate that these neuropeptides have opposing effects on the development of CEH or pyometra in dogs. Our previous study showed significantly higher *Nucb2* expression and nesfatin-1 protein production in overweight bitches and those suffering from CEH or pyometra compared to healthy animals. Additionally, we found that nesfatin-1 immunoreactivity was elevated in diseased uteri of bitches with BCS > 5/9 (28). Moreover, our studies on a group of bitches with CEH or pyometra showed a lower serum phoenixin levels and downregulation expression of this peptide in the uterine tissue of those animals, regardless of their body condition score (BCS). When ovarian cysts and peptide hormones’ association is considered, elevated levels of PNX were reported in female dogs with varying health statuses (26). Similarly, reduced serum phoenixin (PNX) concentration and expression have been observed in women with endometriosis (17). Data on NUCB2/nesfatin-1 expression in PCOS patients are inconsistent, with studies reporting decreases, increases or no change in serum levels (2). Modulation of production of nesfatin-1 in animals with coexisting ovarian cysts and uterus disorders awaits future research.

The presented research indicates that nesfatin-1 expression might correlate with weight gain and potentially impact ovarian and uterine function in dogs. However, it is important to note that the study has limitations, including a small sample size and incomplete data which do not cover all stages of the oestrous cycle. Additionally, differences between dog breeds and factors such as diet and behaviour could have influenced the findings. The study mainly focused on overweight dogs and did not include many obese individuals (BCS > 8/9), and particularly lacked those with metabolic disorders such as hyperglycaemia or diabetes.

## Conclusion

This study is one of the first to demonstrate variations in *Nucb2* mRNA and nesfatin-1 protein expression in female dogs’ reproductive tissues by age and body weight. The study findings indicate increased levels of this neuropeptide and its heightened tissue expression in overweight dogs with a BCS greater than 5/9. This peptide may play a crucial role in regulating energy balance and the ovarian and uterine activity in bitches. However, this hypothesis requires further investigations.
